# Iron and Neurodevelopment in Preterm Infants: A Narrative Review

**DOI:** 10.3390/nu13113737

**Published:** 2021-10-23

**Authors:** Kendell R. German, Sandra E. Juul

**Affiliations:** Department of Pediatrics, University of Washington, Seattle, WA 98195, USA; sjuul@uw.edu

**Keywords:** iron, neurodevelopment, brain development

## Abstract

Iron is critical for brain development, playing key roles in synaptogenesis, myelination, energy metabolism and neurotransmitter production. NICU infants are at particular risk for iron deficiency due to high iron needs, preterm birth, disruptions in maternal or placental health and phlebotomy. If deficiency occurs during critical periods of brain development, this may lead to permanent alterations in brain structure and function which is not reversible despite later supplementation. Children with perinatal iron deficiency have been shown to have delayed nerve conduction speeds, disrupted sleep patterns, impaired recognition memory, motor deficits and lower global developmental scores which may be present as early as in the neonatal period and persist into adulthood. Based on this, ensuring brain iron sufficiency during the neonatal period is critical to optimizing neurodevelopmental outcomes and iron supplementation should be targeted to iron measures that correlate with improved outcomes.

## 1. Introduction

Iron is an essential mineral and iron deficiency is the most common micronutrient deficiency worldwide, affecting approximately 2 billion individuals globally [1]. While the majority of iron deficiency occurs in low-income countries, reproductive-age women and children represent particularly at-risk groups even in middle- and high-income countries [2] and due to decreasing intake of iron-rich foods [3], the rate of iron deficiency within this population may be increasing [4].

The primary role of iron in the body is to facilitate blood oxygen-carrying capacity and delivery. Approximately 70% of iron in the body is found in red blood cells as a component of hemoglobin. In addition to its important role in erythropoiesis, iron also plays key roles in the brain including brain growth, myelination, neurotransmitter production and energy production [5,6]. Based on the critical importance of iron for oxygen delivery, animal studies have shown that iron is prioritized for red blood cell production over other uses for iron, including in the brain [7]. Based on this, changes in red blood cell indices, such as anemia, represent a late marker of iron deficiency. When severe, iron deficiency with inadequate supplementation leads to iron deficiency anemia. The brain, which undergoes rapid development in the neonatal period and is reliant on iron for much of this development, appears to be particularly vulnerable to iron deficiency states. Iron deficiency during key periods of development may lead to disrupted development in areas of the brain that are sensitive to iron status and are undergoing rapid development during these periods. Iron deficiency during these periods (which appear to be the pre-natal and neonatal/infancy period) may have long-lasting neurodevelopmental effects which are not reversible with late supplementation [8,9]. As brain iron stores are depleted before the erythropoietic pool, these neurodevelopmental effects may be seen at iron deficiency levels prior to the emergence of anemia. Conversely, iron excess may also have detrimental effects, highlighting the importance of optimizing iron status, particularly in developing infants.

In this review, we will focus on the importance of iron for brain function and development in the neonatal population. Additionally, we will briefly review key hurdles to overcoming iron imbalance in neonates.

## 2. Iron in Neonates

Maintaining a normal iron balance is particularly important in the neonatal population, and infants are at higher risk than adults for both iron deficiency and overload. Given that the bulk of iron transfer occurs during the third trimester of pregnancy [10], preterm neonates are at particular risk for deficiency if they do not receive adequate supplements. Maternal iron deficiency and conditions that lead to impaired placental function or inflammation, such as diabetes, smoking and obesity, may also lead to impaired fetal stores as iron is actively transported across the placenta [11,12,13]. Iron stores are most critical during the first 4–6 months of life until infants take in iron through supplementary foods, as breastmilk is low in iron (though what limited iron is present is readily absorbed as lactoferrin) [14,15]. Additionally, rapid somatic growth and red cell expansion that occurs following preterm birth leads to increased iron demand in the newborn period. Iron demand is further increased in critically ill neonates due to repeated phlebotomy.

### 2.1. Iron Metabolism

Due to the risks attributable to both iron deficiency and iron overload, iron status is highly regulated in children and adults. Hepcidin, a polypeptide hormone produced in the liver, is the primary regulator of iron absorption and availability. It acts on the transmembrane protein ferroportin. Ferroportin is present on the basal surface of intestinal cells and on cells that commonly store iron such as macrophages and hepatocytes. Ferroportin allows absorption of iron from intestinal epithelial cells into the bloodstream where it is transported to sites of need bound to transferrin. During periods of iron sufficiency, hepcidin values increase. Hepcidin causes the internalization and degradation of ferroportin leading to decreased iron absorption from the gut and sequestration of iron in storage cells such ass hepatocytes and macrophages [16,17]. During periods of iron deficiency or with high iron need, such as during pregnancy, hepcidin is suppressed, thus increasing iron absorption and mobilization [17,18]. The role of hepcidin regulation during pregnancy has been shown to be critical with postnatal iron deficiency being highly correlated with newborn iron values and thus fetal iron loading. Thus, dysregulation of the iron-hepcidin–ferroportin axis in both neonates and pregnant women can significantly impact neonatal iron status and neurodevelopmental outcomes [19,20]. Further, both iron excess and deficiency during pregnancy have been associated with worse neonatal and maternal outcomes [21,22]. For example, maternal anemia has been associated with preterm birth and growth restriction [23,24], highlighting the importance of targeting optimization of iron status in pregnant women to improve neurodevelopmental outcomes of neonates.

Hepcidin levels have also been shown to rise with inflammation [25]. Theoretically this aids to sequester iron from siderophilic bacteria.

Emerging studies have suggested that hepcidin regulation may be intact in the neonatal population [26,27,28].

### 2.2. Iron Supplementation

Due to the high prevalence of iron deficiency worldwide, the majority of research in the field of iron metabolism and supplementation has focused on iron deficiency and iron deficiency anemia. However, free iron is a potential pro-oxidant and iron-mediated ferroptosis has been implicated as a potential cause of brain injury in neonates. Because preterm infants have immature anti-oxidant mechanisms, they may be at particular risk of iron overload. Adverse effects of iron supplementation in iron-sufficient infants and children have been shown, though results are inconsistent and depend upon risk factors of the population. For example, iron supplementation in populations at high risk of malaria has been associated with increased risk of infection, morbidity and mortality from malaria [29,30]. In a prospective study of iron fortification in infancy, Lozoff et al. found that iron-sufficient infants with normal hemoglobin levels who received iron-fortified formula had worse outcomes, compared to the improved outcomes for iron deficient infants in this cohort who received supplements [31]. By contrast, Singhal et al. randomized infants to unfortified cows milk vs. low or high iron-fortified formula in a large cohort in the UK. They did not find any adverse effects on gastrointestinal symptoms, morbidity or infection in those treated with high-iron formula, despite a low prevalence of iron deficiency [32].

Based on this, accurate assessment of iron status is needed to guide iron supplementation, particularly in neonates who are at risk for both iron deficiency and toxicity, described by Brannon and Taylor as a U-shaped risk curve for iron in this population [33].

Due to the risks attributable to iron deficiency and the increased prevalence of low iron stores in the preterm population, many regulatory bodies recommend iron supplementation in this population, though the amount of iron that optimizes outcomes is not clear and supplementation guidelines are not uniform throughout different organizations. The American Academy of Pediatrics recommends that preterm infants receive 2 mg/kg/day of iron supplementation beginning in infancy and that exclusively breastfed infants receive 1 mg/kg/day of iron supplements beginning at 4 months of age [34,35]. Similarly, the European Society of Pediatric Gastroenterology, Hepatology, and Nutrition (ESPGHAN) recommends that breastfed, preterm infants receive 2–3 mg/kg/day of iron supplements through 6 months of life [36]. By contrast, the United States Preventative Services Task Force finds insufficient evidence to recommend routine supplementation of iron [37]. However, emerging research in the field of iron metabolism suggests that 2 mg/kg/day may be insufficient to replenish iron stores in high-risk populations such as preterm neonates and enteral supplementation doses up to 12 mg/kg/day have been used in several prospective studies without noted ill effects [38,39]. In fact, improved developmental scores have been seen with higher doses of supplementation in limited studies [40]. Ongoing research is aimed at evaluating optimal doses of iron supplementation (NCT04691843).

### 2.3. Monitoring Iron Status

Due to the risks of both iron deficiency and overload, careful regulation of supplementation is likely needed to ensure optimal iron balance. However, due to the normal hematologic changes of infancy, frequent transfusions and inflammatory conditions, accurate assessment of iron status can be challenging in neonatal populations. Table 1 summarizes the iron indices most commonly used in this population, with the advantages and disadvantages of each measure and their relation to neurodevelopment highlighted. Based on currently available evidence, a combined approach is likely needed.

### 2.4. Iron and Neurodevelopment

The association between iron deficiency and adverse short-, medium- and long-term outcomes has been widely studied in both animal models and in human infants and children. Iron deficiency during the neonatal period has been consistently associated with worse neurodevelopmental outcomes broadly, with specific areas of deficit related to brain regions that appear to be most affected by iron deficiency, such as the hippocampus and myelination [53,54,55]. Unique to infants, brain iron deficiency is induced more quickly in the setting of iron deficiency and disruptions in development caused by iron deficit are not reversible with later repletion [56], which makes ensuring adequate brain iron status particularly critical in the neonatal period.

Animal models have elucidated both biochemical and structural effects of iron deficiency on the brain and demonstrated that susceptibility periods to iron deficiency vary over time and in different areas of the brain [57]. The hippocampus is a highly metabolically active part of the brain that undergoes rapid development in the neonatal and toddler period. Rodent and primate models of iron deficiency demonstrate less complex dendritic arborization [58], alterations in synaptic plasticity and regulation [59], impaired dopamine-mediated neurotransmission [56] and persistent metabolic disruptions [60]. Through the use of knockout mice to prevent iron utilization in the brain without anemia, these models are able to specifically demonstrate that these findings are attributable to iron deficiency rather than anemia, allowing the isolated impact of iron deficiency (versus anemia and impaired oxygen-carrying capacity) to be determined [59].

Neurodevelopmental effects of iron deficiency can be seen early in the neonatal period. In children, one tool for assessing short-term brain changes includes the Auditory Brainstem Response (ABR), a test which correlates with neuronal transmission speed and therefore myelination. Amin et al. showed shorter inter-wave latencies in iron-replete versus -deplete newborns [61]. Similarly, Berglund et al. found that iron supplementation correlated with shortened inter-wave latencies on ABR [62]. Algarin et al. showed that differences in ABR latencies persisted into early childhood, with children with a history of iron deficiency anemia showing longer inter-wave latencies at 4 years of age [63]. Even changes in infant temperament and reflexes can be noted in the neonatal period in association with iron status [64,65].

Medium-term studies have examined neurodevelopmental outcomes of children with perinatal iron deficiency in toddlerhood and early school age. These studies have shown a wide range of neurodevelopmental effects associated with low iron status and/or low iron supplementation including lower language skills [19], higher inattention scores [19], disruptions in sleep patterns [66], impaired recognition memory [67] disrupted motor development [19,68], slower processing speed [68], and social interaction differences [68].

Dr. Lozoff and her colleagues have followed long-term cohorts of children from Chile and Costa Rica, evaluating associations between iron status in infancy and developmental measures from infancy through early adulthood [69,70,71,72,73]. Her group has found that young adults with a history of iron deficiency in infancy have worse executive function skills, recognition memory and cognitive scores. Additionally, iron deficiency in infancy was found to be associated with increased inattention and lower verbal IQ scores in childhood and lower educational attainment in adulthood, which they postulate may be related to the impacts of iron deficiency in childhood [70]. Impacts on self-reported emotional health were also observed [71]. However, studies by this group also show that universal iron supplementation in formula may have detrimental effects highlighting the importance of targeting supplements to need [74,75]. Additional long-term studies include a potential association seen between maternal anemia (felt likely to be secondary to iron deficiency) and increased risk of psychiatric disorders in offspring [76,77], though these studies are observational in nature therefore results must be interpreted with caution. Taken together, there is evidence that iron deficiency in the perinatal period has the potential to effect long-term change into adulthood further emphasizing the importance of this critical window.

Although iron deficiency in the neonatal period is of the most significant importance, later iron deficiency does have cognitive and other neurophysiologic effects. Even in adults who develop iron deficiency, performance differences can be seen [78], though iron overload is associated with neurodegenerative conditions [79], emphasizing the importance of maintaining a normal iron balance throughout an individual’s lifespan.

Conversely, iron overload may also have neurodevelopmental effects, though fewer studies exist in the literature examining the long-term effects of iron overload on preterm infants. This skew may reflect either that iron deficiency versus overload is of greater concern to this population or may reflect a bias in the field [80]. Free iron, through production of hydroxyl radicals via the Fenton reaction, is a pro-oxidant which may lead to direct cellular injury through lipid peroxidation. Preterm infants, who have immature anti-oxidant mechanisms and high transferrin saturation levels, may be at particular risk to free radical-mediated cell injury [81]. Oxidative stress has been implicated in several diseases of prematurity, including retinopathy of prematurity and chronic lung disease. Iron-rich transfusions have been associated with these disease conditions, though these studies suffer from the confounding effects of transfusions’ activation of the inflammatory cascade and higher use in sicker preterm infants which limits our ability to understand the direct role of iron on these conditions [82,83,84].

Free iron released from brain hemorrhage is clearly associated with neonatal brain injury through ferroptosis (i.e., iron-mediated cell death). Hemin, which is produced from hemoglobin breakdown and leads to release of Fe2+ and Fe3+, induces astrocyte injury in mouse models through free radical production leading to lipid peroxidation [85] and protein and DNA injury [81]. This iron-mediated cascade leads to a “second hit” injury in neonates who suffer intraventricular hemorrhage, with an initial insult related to the hematoma itself and a secondary injury caused by iron-mediated ferroptosis. The role of iron in neonatal brain injury is suggested by studies which show protective effects of iron chelators and erythropoietin [86]. However, it is unclear whether non-heme-derived iron has the same detrimental effect [85]. Some studies have shown that elevated iron predates intraventricular hemorrhage, suggesting that elevated iron may play a role in brain injury, though these studies are limited in size, thus making definitive conclusions difficult [87]. Additionally, markers of oxidative injury do not appear to be associated with enteral iron supplementation [88], though studies are mixed [89]. This may suggest that enteral iron does not have the same toxicity risks in comparison with iron released from hemorrhagic injury, though further studies are needed. Any discrepancy may be due to the regulatory effects of hepcidin, which help prevent the absorption of excess iron and appear to be functional in the preterm population [26,28,90] or due to the protective effects of lactoferrin in infants fed human milk, which may protect against iron-mediated lipid peroxidation [91].

Long-term neurodevelopmental effects that are directly attributable to iron overload are unclear [92]. Iron overload is thought to underlie many neurodegenerative diseases such as Alzheimer’s disease and Parkinson’s disease. Animal studies have suggested a potential risk for these effects based on high-dose supplementation in infancy, but to our knowledge this effect has not been reported in the human setting [93].

## 3. Recommendations and Future Directions

Despite considerable research focused on the importance of iron status and its impacts on brain development, the optimal iron supplementation dose, monitoring regimen, and target markers are still not clear. Because of this, the most important component of iron regulation in neonates is to avoid the development of iron imbalance through minimizing maternal iron deficiency, optimizing maternal health during pregnancy, and preventing neonatal phlebotomy.

To address ongoing gaps in our knowledge of neonatal iron homeostasis, the evaluation of iron marker set-points that correlate with non-hematologic outcome measures would be of significant benefit [33]. Tamura et al. found that infants with ferritin values in the lowest quartile, <76 microgram/L, were associated with worse neurodevelopmental outcomes [19]. Based on this, we would propose that ferritin values above this range are likely needed to ensure adequate iron is available for brain development. However, optimal cutoffs for ferritin and other iron markers, that maximize outcomes have not been established.

Phlebotomy in critically ill neonates is a primary contributor to iron deficiency. Measuring iron status through serum measures may potentiate these losses. Emerging studies have started to evaluate non-hematologic measures of iron status, such as urine ferritin or urine hepcidin [28,90,94]. Additionally, measures such as reticulocyte hemoglobin equivalent, which is measured in conjunction with a complete blood cell count on many analyzers, may be of benefit, though more robust assessment of goal values that optimize outcomes in critically ill neonates are needed.

Finally, we are beginning to recognize the critical importance of the gut microbiome on health. Of particular relevance to this review, is the association between the gut bacterial population and neurodevelopment, with high levels of Bacteroides species associated with improved developmental outcomes [95]. Although the exact mechanism through which the microbiome affects neurodevelopment is unclear, it has been well documented that dysbiosis is correlated with neurodevelopmental effects. When considering enteral iron supplements, although regulation of iron absorption through hepcidin [96] may protect infants against iron toxicity when supplements are administered enterally, high levels of unabsorbed iron in the gut lumen may have significant impacts on microbial populations and gut inflammation [97,98]. Specifically, potentially pathogenic species, such as Clostridia, E. coli and Pseudomonas are thought to expand in the presence of increased luminal iron [99,100], suggesting that excessive enteral iron supplementation is not without risk, particularly in preterm infants who are already at increased risk for dysbiosis and intestinal inflammation. Further studies are needed to identify ways to balance iron supplementation with microbiome health through alternative formulations and potentially intravenous preparations.

## 4. Conclusions

In summary, optimizing iron balance is an important component of neurodevelopmentally-focused neonatal critical care as it represents a common yet potentially modifiable intervention. While iron excess should be avoided, there is sufficient evidence to support iron supplementation in infants with iron deficiency in order to prevent anemia and ensure that brain development is not interrupted. However, there is likely no universal supplementation dose that is appropriate for all neonates since infants have variable risk factors both pre and postnatally. By contrast, an individualized supplementation strategy based on iron measures is needed in at-risk infants. Future national and other expert guidelines would benefit from recognition of the need for individualized dosing guidelines in preterm neonates and ideally individualized strategies based on iron measure targets that are based on neurodevelopmental outcomes. Ongoing research in this field has the potential to improve outcomes in both high- and low-resource settings.

## Figures and Tables

**Table 1:** Commonly used iron measures in neonates.

Iron Measure	Description	Advantages	Disadvantages	Association with Neurodevelopment
**Ferritin**	Storage form of iron	Most widely studied marker of iron status in the neonatal population. Low ferritin values are a reliable marker of iron status.	Elevated in the setting of inflammation and hepatocellular injury [41].	The association between ferritin values and neurodevelopmental outcomes has been studied [19]. Low ferritin values are associated with worse outcomes, but high values may be less reliable due to inflammatory effects [42].
**Zinc Protoporphyrin-to-Heme Ratio (ZnPP/H)**	Zinc or iron can be incorporated into the protoporphyrin ring in the production of heme. When less iron is available, the proportion of zinc incorporated increases, thus raising the ZnPP/H ratio.	Less affected by inflammation [39]. Has been evaluated in neonatal and childhood population [43,44,45,46].	ZnPP/H values in transfused adult red blood cells may dilute neonatal values in infants who have received transfusions. Reflect values in the red blood cell pool as a whole, and therefore acute changes in iron status may not be reflected until older red blood cells are broken down [47].	May be better correlated with neurodevelopmental outcomes than ferritin, though studies are limited [42].
**Reticulocyte Hemoglobin Equivalent (Ret HE)**	The hemoglobin content of reticulocyte cells.	As it reflects newly formed cells, it may be more responsive to recent changes in iron status. Adult studies suggest reliable with inflammation [48].Can be measured in conjunction with a complete blood cell count (CBC) on many CBC analyzers, thus limiting phlebotomy.	Although some studies have begun to establish normative values in the neonatal population, there are limited studies addressing its correlation with long-term outcomes and therefore target values are still unclear [49,50].	Limited data examining correlation with neurodevelopmental outcomes.
**Hemoglobin/Hematocrit**	The majority of iron in the body is present in red blood cells.	Readily available and low cost.	As iron is prioritized for erythropoiesis over all other needs, anemia is a late marker of iron deficiency and adverse impacts such as brain iron deficiency may occur in the absence of anemia.	Studies examining correlation with outcomes may reflect iron deficiency status or non-iron factors such as transfusion thresholds [51,52].
**Transferrin Saturation**	Transferrin is a protein that transports iron around the body. The transferrin saturation reflects the percentage of transferrin sites that are occupied by iron.	Can be calculated from TIBC and serum iron.	Normative values not well defined in neonates.	Limited data examining correlation with neurodevelopmental outcomes.
**Total Iron-Binding Capacity (TIBC)**	A measure of the level of transferrin in circulation.	Commonly used measure in adults.	Normative values not well defined in neonates.	Limited data examining correlation with neurodevelopmental outcomes.

## Data Availability

Not applicable.
